# Errata

**Published:** 1894-01

**Authors:** 


**Errata.**—"Accidents will happen in the best regulated" business. Typographical errors are "accidents." It is almost impossible to prevent them with our present system of type-setting and printing,—although the editor cheerfully owns up to at least a part of the fault—whereby such errors happen; for instance, in Dr. Moss' splendid article in our December No.—"Dench apparatus" was set up and printed "*douch apparatus.*" Now, that is very provoking, and is one instance where the editor is to blame; he ought to have had better sense than to let a "proof" go, in which it was recommended to "douche" fumes of camphor, etc., through a catheter in treatment of eustachian and middle ear trouble—he ought to have readily recalled his old friend (?) "Dench" and his apparatus in that connection, but he didn't; but the fact is—all editors are not perfectly familiar with the names of all the inventors of eye and ear instruments, and other inventors, and instruments named for them, their name is (not Denis) "legion!" Then, again, authors of papers should write the names of all such plainly. They all seem to think an editor knows as much as they do, and is acquainted with all their text books. But,

What we started to say is—

On page 274, December No., near top of page, for "*douch apparatus*" read "Dench apparatus"; also, in last case reported, for December 10, 1893, read December 10, 1892.

In Dr. Bibb's article (in November No.), for "vaccinæ," read "vaccinal," and for "ante-intoxication", read "acute intoxication"; for "gayical" read "guaiacal," and for "report" read "attempt." There; we feel better.

